# Kinetic changes in sweat lactate following fatigue during constant workload exercise

**DOI:** 10.14814/phy2.15169

**Published:** 2022-01-19

**Authors:** Hiroki Okawara, Tomonori Sawada, Daisuke Nakashima, Yuta Maeda, Shunsuke Minoji, Takashi Morisue, Yoshinori Katsumata, Morio Matsumoto, Masaya Nakamura, Takeo Nagura

**Affiliations:** ^1^ Department of Orthopaedic Surgery Keio University School of Medicine Shinjuku Japan; ^2^ Institute for Integrated Sports Medicine Keio University School of Medicine Shinjuku Japan; ^3^ Department of Cardiology Keio University School of Medicine Shinjuku Japan; ^4^ Department of Clinical Biomechanics Keio University School of Medicine Shinjuku Japan

**Keywords:** fatigue, lactic acid, physiology, sports, sweating

## Abstract

It is useful to investigate various physiological responses induced by fatigue in athletes. Moreover, wearable noninvasive sensors, including sweat sensors, are compatible with fatigue evaluation because of their ease of use, and ability to measure repeatedly and continual data. This cross‐sectional study aimed to clarify how sweat lactate elimination curves obtained during constant workload exercise changed following fatigue. Seventeen recreationally trained males (average age, 20.6 ± 0.8 years) completed two consecutive constant workload exercise tests (at 25% peak power) with rest intervals; the participants were encouraged to perform Test 1 until exhaustion and Test 2 only for 10 min. Subjective fatigue (numerical rating scale with face rating scale), sweat lactate, and sweat rate were measured for 10 min in each test. Subjective fatigue was compared using the Wilcoxon signed‐rank test and time to each constant value between Tests 1 and 2 was compared using paired *t*‐test. Subjective fatigue significantly increased during Test 2 compared with that during Test 1. After Test 1, the sweat lactate elimination curve demonstrated a leftward shift, as proved by the significantly sooner observation of the peak and constant values of sweat lactate (2, 3, and 4 μA) (*p* < 0.01). Our preliminary results suggest that the sweat lactate elimination curve is different in the fatigue state. Further research may provide insight in the application of this curve to the evaluation for fatigue.

## Graphical abstract

To evaluate the changes in sLA after fatigue using a wearable sLA sensor, comparing the sweat lactate elimination curve during constant workload exercise between in fatigued state and notfatigued in twenty healthy recreationally trained males was performed. As a results, a leftward shifted curve was detected, which suggest the possibility of taking insight in the application of this curve to the evaluation for fatigue by further research.

## 1 INTRODUCTION

Recently, wearable or attachable biosensing and analytical software advances have provided considerable possibilities to evaluate and estimate physiological information using data obtained from the body surface at medical, healthcare, and sports facilities. For example, parameters including glucose (Karpova et al., 2019; Kudva et al., 2018; Nesaei et al., 2018; Wiorek et al., 2020), cortisol (Parlak et al., 2018), endurance performance (Shiraishi et al., 2018), core body temperature (Matsunaga et al., 2020), and nutrition level (Zhao et al., 2021) can be evaluated. Of the various biomarkers targeted for biosensing, sweat is a preferable choice because sweat is readily accessible and contains important electrolytes, metabolites, amino acids, proteins, and hormones. In particular, sweat lactate (sLA) measurement has some advantages over other alternative methods, such as ease of use, noninvasiveness, and the ability for continual measurement. In addition, although some researchers have concluded that sLA only exhibits the metabolism of sweat glands (Baker & Wolfe, 2020; Derbyshire et al., 2012) and gave no insight into the clinical use of sLA, it has been reported that continual monitoring of sLA provides new information that is considered to respond to physical conditions using the sLA curve (Katsumata et al., 2021; Seki et al., 2021). Therefore, there has been a large increase in the use of wearable sensors to measure sLA recently (Currano et al., 2018; Gao et al., 2016; Kai et al., 2018; Luo et al., 2018; Vinoth et al., 2021).

Years of sports science research have brought great progress in improving the performance of the athlete, for example, harder collisions, higher jumps, or longer and faster runs in various sports. However, greater intensity in these activities has also led to a higher level of physical demand and burden for athletes (Stølen et al., 2005; Tavares et al., 2017). This may in turn lead to an excessive level of accumulated fatigue in athletes during the match or training. In addition, the interruption of sport events because of the COVID‐19 pandemic has led to shortened match intervals and insufficient recovery time for athletes, aggravating the fatigue condition (Mota et al., 2020). Because fatigue is strongly associated with injury (Jones et al., 2017), athletes, their coaches, and medical staff need to monitor it carefully. Thus, it is crucial to investigate the physiological responses of athletes to fatigue. A previous report has demonstrated the effect of fatigue on creatine kinase (Meyer & Meister, 2011), cortisol testosterone (Hough et al., 2013; Kraemer et al., 2004), and amylase (Nater & Rohleder, 2009) levels. These physiological stress markers, particularly those obtained from the saliva, have an advantage in terms of simplicity. However, there are limitations with regard to the cost and time required for analysis. In addition, some of these markers are often affected by psychological stress (Alix‐Sy et al., 2008) and circadian rhythm (Lac, 2001; Minetto et al., 2008; Nater & Rohleder, 2009), and have large individual variability (Hartmann & Mester, 2000; Twist & highton, 2013).

To overcome these limitations, wearable sensors offer various advantages and are ideal for evaluating the physiological responses to fatigue. However, to the best of our knowledge, there are no reports on the use of wearable sensors for the evaluation of fatigue caused by exercise. Among the various wearable sensors, the sLA sensor is suitable because of the accessibility of sweat during exercise. Therefore, we performed this study to evaluate the changes in sLA after fatigue using a wearable sLA sensor.

## 2 MATERIALS AND METHODS

### 2.1 Participants

Twenty healthy recreationally trained males with an average age of 20.6 years participated in this study between May and August 2020. The inclusion criteria were as follows: (1) age between 18 and 25 years and (2) regular exercise more than three times per week. The exclusion criteria were as follows: (1) lower extremity injury or disorder that disturbs complete participation in exercise, (2) metabolic, cardiac, respiratory, and psychiatric diseases and (3) habitual smoking. We certify that all applicable institutional and governmental regulations concerning the ethical use of human volunteers were followed throughout this research. The study protocol was conducted in compliance with ethical guidelines for medical and health research involving human subjects and was approved by the P‐One Clinic Ethical Committee and the Ethics Committees of our institution (Approval no. 20190357). Written informed consent was obtained from the study participants for study participation and publishing of the findings before enrollment.

### 2.2 Protocol

We performed this cross‐sectional study to verify the influence of fatigue on sLA in a constant‐load exercise test at sports facilities according to a previous study (Sassi et al., 2006). Participants underwent all tests without intake of any food for 3 h (Lamont, 1985; Pöchmüller et al., 2016); intake of caffeine for 12 h (Wiles et al., 1992); and alcohol consumption and hard activity for 24 h.

**FIGURE 1:**
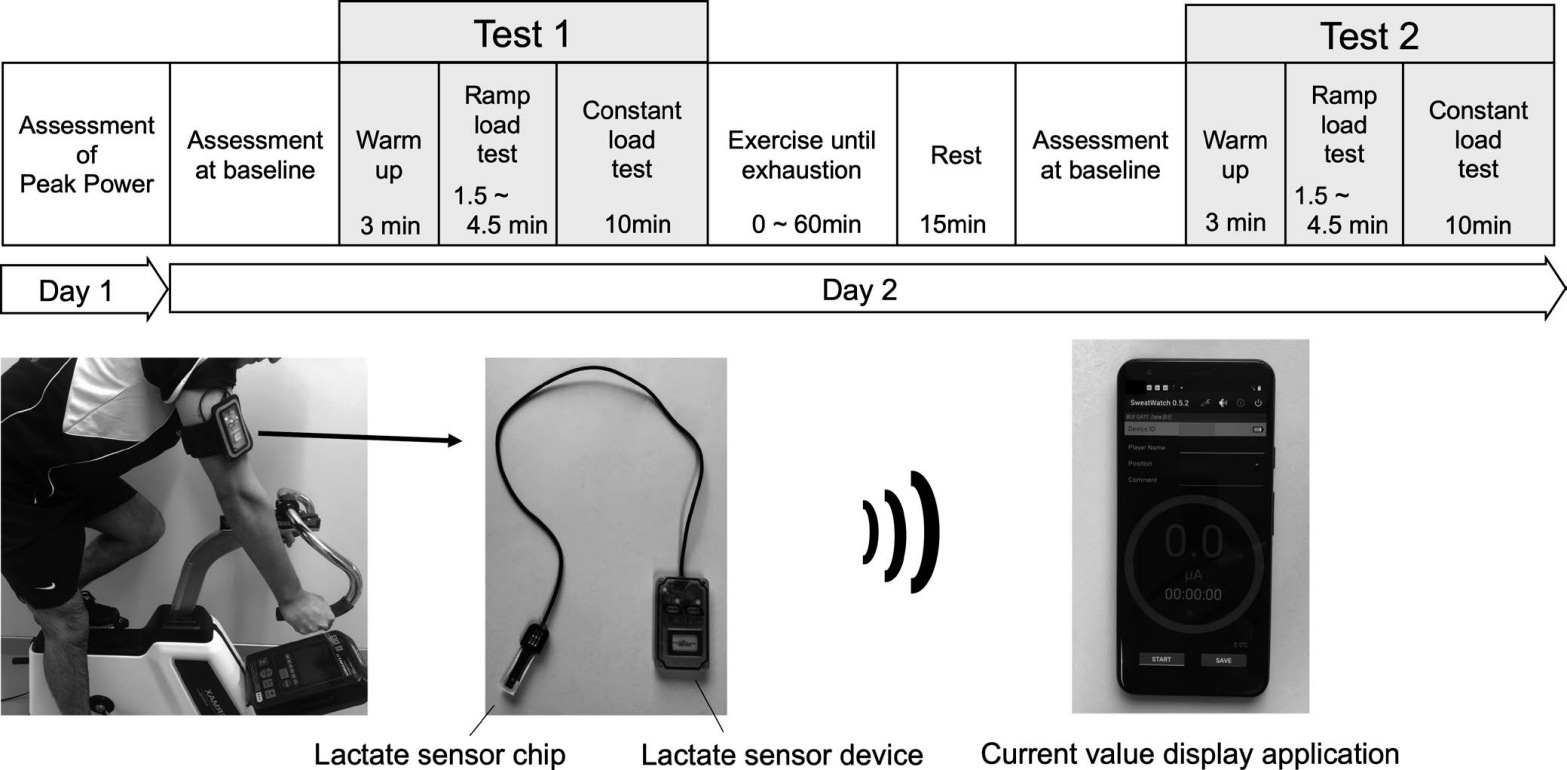
Experimental procedure to evaluate sweat lactate using lactate sensor device and sensor chips. This figure shows the experimental procedure and a set of devices used to evaluate sweat lactate. It was defined as leaving more than 7 days between days 1 and 2. The data were recorded on a mobile application through a Bluetooth connection. The sensor chips were replaced during the rest phase between Tests 1 and 2

All the test flows are shown in Figure 1. Peak power (PP) was evaluated by adapting the Wingate test with an applied resistance of 11% of body weight (Bar‐Or, 1987; Jaafar et al., 2014) using an electromagnetically braked ergometer (POWER MAX V3 Pro, Konami Sports Co., Ltd.) approximately 1 week before the exercise test day.

On the exercise test day, each participant exercised twice on the cycle ergometer with a constant workload of 25% PP. Based on the results of preliminary experiment performed at our laboratory refer to previous study (Sassi et al., 2006), this workload was selected to lead the subject to exhaustion within 10–70 min. After 1‐min warm‐up at 50 W, the main test was conducted, through which the cadence rate of pedaling was set to 70–80 revolutions per minute (rpm). The target constant workload was gradually reached with increments of 20 W every 30 s to avoid a sudden increase in sLA. As this protocol was based on one used in a previous study (Sassi et al., 2006) which included participants of a higher level of athletic ability than those in our study, we adopted a gentler way of increasing the workload. After reaching the target load, participants were encouraged to complete the exercise to exhaustion, which was defined as an unsustainable state to pedal at over 70 rpm (Test 1). Following exhaustion, the participants were asked to rest for 15 min; following this, they completed the same exercise as Test 1 for 10 min with the same constant workload (Test 2). Throughout these experiments, the room temperature was maintained between 24 and 26°C and humidity was maintained between 45%–65%.

### 2.3 Outcomes

#### 2.3.1 Body composition

The body composition was assessed using a multifrequency segmental body composition analyzer (MC‐780A‐N, TANITA Co., Ltd.) before Test 1.

#### 2.3.2 Sweat lactate and sweat rate

To evaluate sLA levels, we used a set of wearable sLA sensor devices and sensor chips (Grace Imaging. Inc.) as shown in Figure 1. The sensor device quantifies the lactate concentration as a current value because the chip reacts with sLA and generates an electric current (Seki et al., 2021). The current value can be obtained as continuous data within 0.1–80 μA, with 0.1‐μA increments.

After calibration using saline, the sensor chip which sensor device connected to was attached to the superior right upper limb and forehead, which was cleaned with an alcohol‐free cloth, of the participants. We assessed the sLA from baseline to 10 min after the start of the constant workload exercise in each test. The sensor chips were replaced during the rest period, in consideration of the effect caused by slight incremental dislodgment of lactate oxidase in the sensor chip when in prolonged contact with sweat. In addition, the data were recorded with a sampling frequency of 1 Hz for mobile applications (Grace Imaging. Inc.) using a Bluetooth connection. The recorded data were converted to moving average values over 13 s intervals and individually underwent zero correction using the baseline value. Additionally, these data were multiplied by the sweat rate measured in the same area using a perspiration meter (SKN‐2000M; SKINOS Co., Ltd.) to determine the elimination value per unit area (Hirakawa et al., 2021; Jung et al., 2018).

#### 2.3.3 Numerical rating scale with a face rating scale for fatigue

The objective fatigue intensity was measured using a numerical rating scale supplemented with a face rating scale (NRS‐FRS) with high validity and reliability (Chuang et al., 2015). This combination scale, comprising a numeral rating scale which evaluates individuals’ fatigue level on a scale from 0 to 10 (with “0” indicating “no fatigue” and “10” indicating “worst possible fatigue”) and six‐face rating scale, allows for accurate assessment of fatigue intensity even in the participants with fatigue‐related cognitive function impairment. NRS‐FRS was assessed at baseline and at 1.25, 2.5, 3.75, 5, 7.5, and 10 min after starting the constant workload exercise during each test.

### 2.4 Statistical analysis

As an ordinal scale, we used the Friedman's test and the Bonferroni method to compare NRS‐FRS within each of the two tests and the Wilcoxon signed‐rank test to compare NRS‐FRS at every measurement time between Tests 1 and 2. As the effect size, we calculated *η* ^2^ using *X* ^2^ for the Friedman's test (Morse, 1999) and *r* using *z*‐score for the Wilcoxon signed‐rank test (Field, 2018). To evaluate the shift in the sLA curve, the time from the start of constant workload exercise until the time at which the value of sLA reached a certain value (1, 2, 3, 4 μA, and peak value) was calculated as the time to each constant sLA value. Based on the pre‐performed Shapiro–Wilk test, a paired *t*‐test was applied to compare the time to each of the four constant values of sLA between Tests 1 and 2. We calculated Cohen's *d* using the value of *t* for the paired *t*‐test (Field, 2018). Data were analyzed using IBM SPSS Statistics (version 26.0; IBM Corp.), with statistical significance set at 0.05.

## 3 RESULTS

### 3.1 Participant characteristics

In all, 17 participants who met the inclusion criteria completed this study protocol. Three participants were excluded as they could not continue the exercise for 10 min in Test 2 because of fatigue. The characteristics and body composition data of the remaining 17 participants for the final analysis are shown in Table 1, and individual data of PP are shown in Table S1.

**TABLE 1:** Characteristics of study participants

Variable	Mean (SD)	Range
Age, years	20.6 (0.8)	19–22
Height, m	171.4 (5.5)	163.1–181.9
Weight, kg	63.0 (7.6)	47.1–75.9
BMI, %	21.4 (1.8)	17.7–23.7
Body fat ratio, %	16.2 (4.0)	7.5–24.8
Fat mass, kg	10.4 (3.4)	3.5–17.7
Lean body mass, kg	52.8 (4.9)	43.5–60.6
Muscle mass, kg	49.8 (4.7)	41.2–57.5
Total body water, kg	36.3 (3.8)	29.2–41.7
Body water, %	57.4 (4.5)	49.9–65.4

Note

This table shows the baseline characteristics of the 17 study participants. Body composition data were obtained using a multifrequency segmental body composition analyzer before Test 1 on the second day of the visit.

### 3.2 Fatigue

**FIGURE 2:**
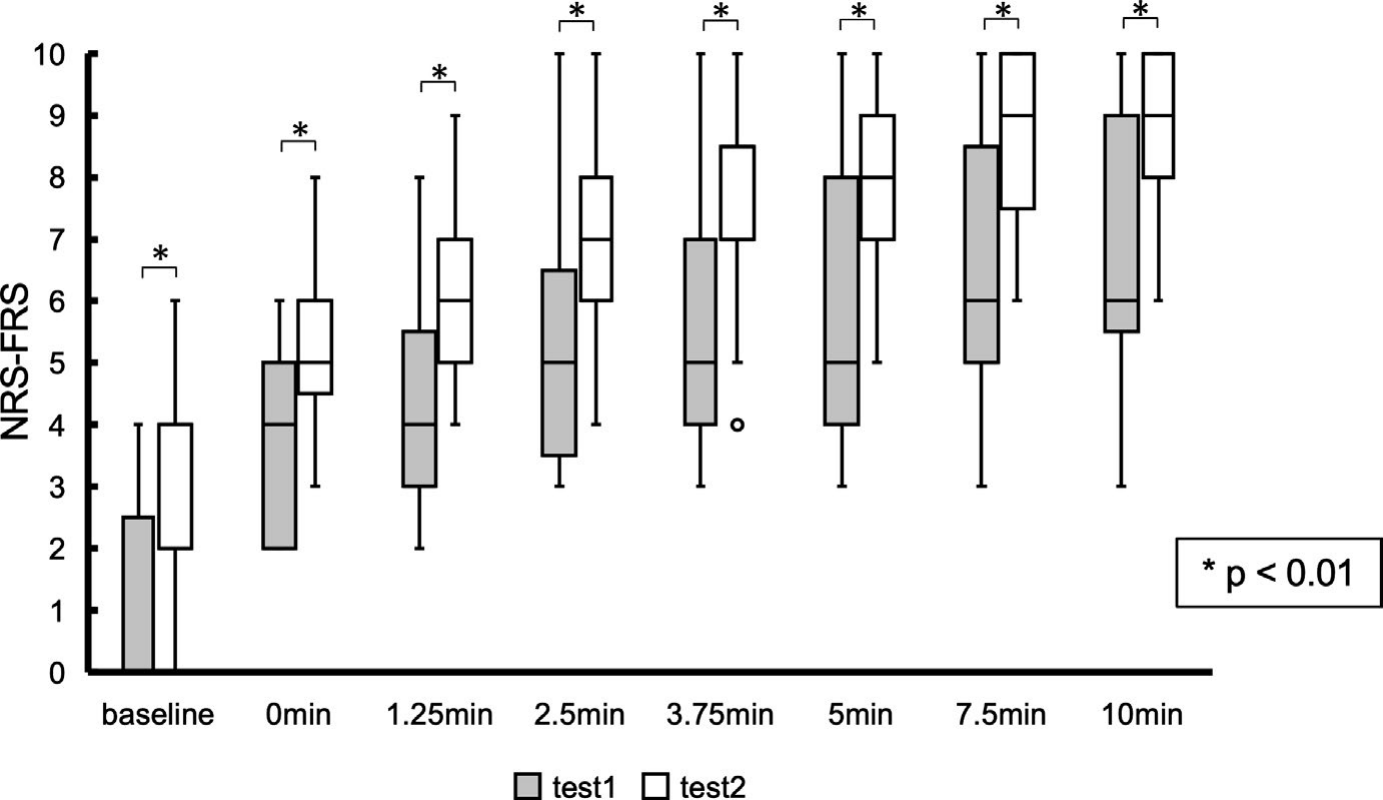
Changes in objective fatigue from Test 1 to Test 2. This figure shows the changes in the numerical rating scale with the face rating scale for fatigue between Test 1 and Test 2. Each value at every time point significantly increased from Test 1 to Test 2. This result indicates that constant workload exercise in Test 1 causes exhaustion; *p < 0.01, using the Wilcoxon signed‐rank test. NRS‐FRS, numerical rating scale with face rating scale

During each test, NRS‐FRS scores significantly increased gradually over time during the constant workload exercise (Test 1: *p* < 0.01, *η* ^2^ = 0.93, Test 2: *p* < 0.01, *η* ^2^ = 0.93, Figure S1). NRS‐FRS scores at every time point in the constant workload exercise increased significantly between Test 1 and Test 2 (Figure 2). The medians (the first quartile and the third quartile) were 0 (0, 2) and 4 (2, 4) at baseline (*p* < 0.01, *z* = −3.27, *r* = 0.79); 4 (2, 5) and 5 (5, 6) at the start of the constant‐load exercise (*p* < 0.01, *z* = −3.23, *r* = 0.81); 5 (4, 8) and 8 (7, 9) at 5 min (*p* < 0.01, *z* = −2.95, *r* = 0.71); and 6 (6, 9) and 9 (8, 10) at 10 min (*p* < 0.01, *z* = −2.70, *r* = 0.65), respectively.

### 3.3 Shift in sLA curve

**FIGURE 3:**
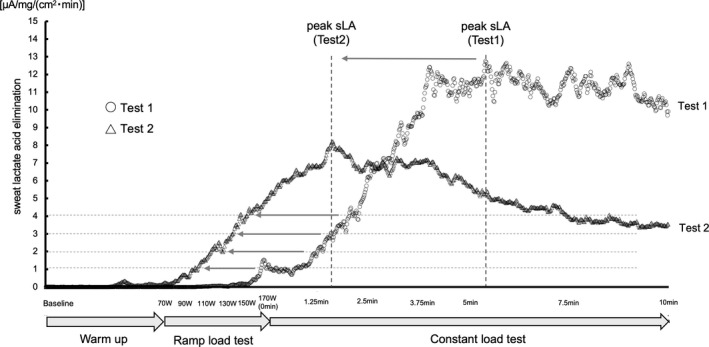
Shift in sweat lactate elimination per unit area curve of representative data after fatigue. This figure shows representative graphs of sweat lactate elimination per unit area curves obtained in Tests 1 and 2. The timing when the value of sweat lactate elimination per unit area reached a certain value (2 μA/mg/(cm^2^ × min), 3 μA/mg/(cm^2^ × min), 4 μA/mg/(cm^2^ × min), and peak value) is shown as triangles (Test 1) and circles (Test 2), and are used for calculating the time to reach each constant value of sweat lactate. sLA, sweat lactate

**FIGURE 4:**
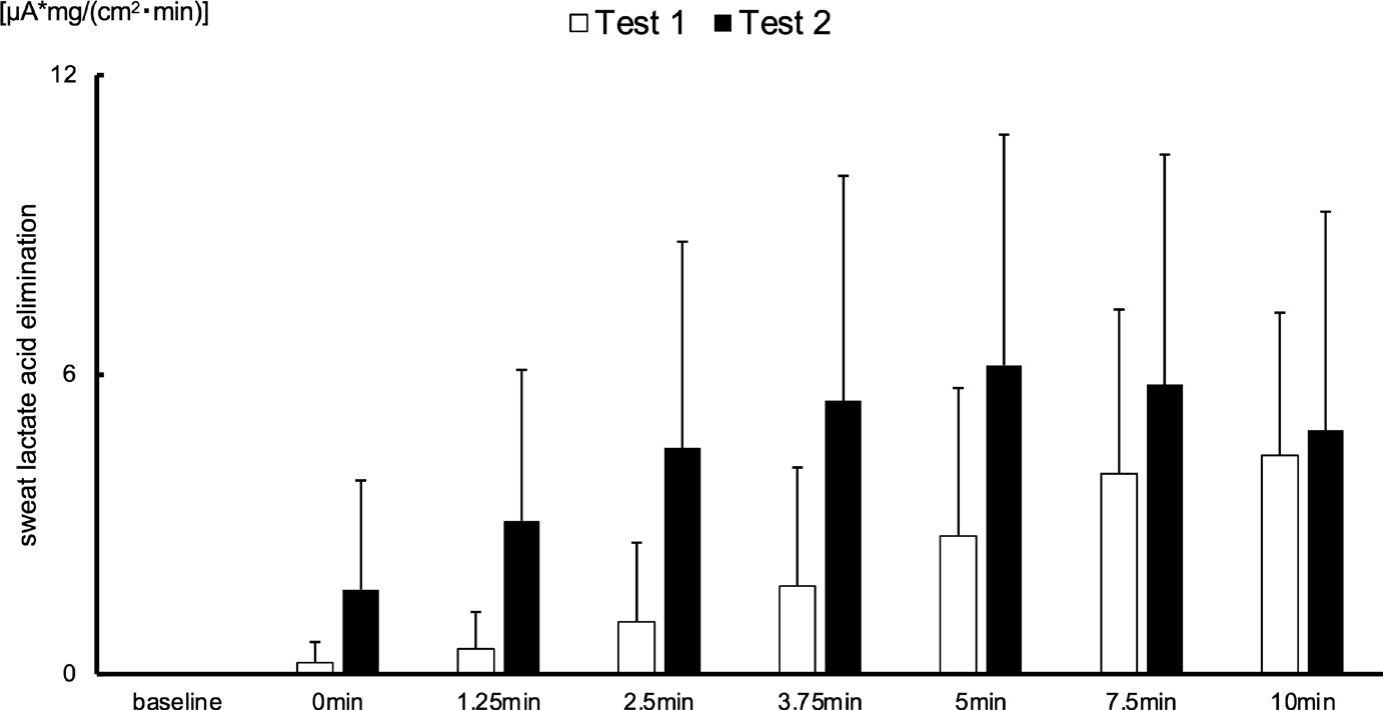
The trend observed for sweat lactate elimination per unit area (on the arm) during pedaling exercise at constant load before and after fatigue. This figure shows the average data of sweat lactate elimination per unit area on the arm at each time point during pedaling exercise with constant workload in Tests 1 and 2. Sweat lactate reached the peak value sooner in Test 2, contrary to the trend of linear increase in Test 1

Representative data of sLA in Tests 1 and 2 are shown as the amount of elimination per unit area in Figure 3 (simple sLA and sweat rate data are shown in Figures [Link], [Link], [Link], [Link]). On overlapping the sLA curves in Tests 1 and 2, the sLA curve was observed to have shifted leftward from Test 1 to Test 2. The mean values of sLA at all measurement time points in Tests 1 and 2 are shown in Figure 4. While sLA demonstrated a linear increase over time in Test 1, it reached the peak value sooner in Test 2, similar to the result obtained with the head measurements (Figure S6). Meanwhile, the sweat rate continued to rise in both tests, unlike sLA (Figures S5 and S6). There was a significant change in the time to the peak value of sLA between Test 1 and Test 2 (Table 2). Similar results showing sLA reaching 1, 2, 3, and 4 μA values at significantly sooner time points in Test 2 compared with Test 1 are exhibited in Table 2. Accordingly, all changes in time for sLA reaching a certain value between Test 1 and Test 2 indicated that the sLA curve was shifted to the left on the *x*‐axis, displaying a time course with a large effect size.

**TABLE 2:** Time to each constant value of sweat lactate elimination per unit area before and after fatigue

	Test 1	Test 2	Mean difference (95% CI)	Cohen's *d* (95% CI)	*p* value*
Time to peak value (sec)^a^	822.1 ± 119.2	671.8 ± 143.9	150.3 (77.2, 223.4)	1.06 (0.45, 1.65)	<0.01
Time to 1 μA/cm^2^ (sec)^b^	578.4 ± 214.1	335.3 ± 167.6	243.1 (171.4, 314.7)	1.74 (0.97, 2.50)	<0.01
Time to 2 μA/cm^2^ (sec)^c^	590.1 ± 154.7	352.6 ± 172.0	237.5 (158.2, 316.8)	1.73 (0.88, 2.56)	<0.01
Time to 3 μA/cm^2^ (sec)^d^	640.3 ± 160.2	372.8 ± 114.8	267.5 (206.0, 329.1)	2.63 (1.45, 3.79)	<0.01
Time to 4 μA/cm^2^ (sec)^e^	678.7 ± 159.8	410.8 ± 129.4	267.9 (196.3, 339.5)	2.51 (1.27, 3.73)	<0.01

Note

This table shows the changes in the times taken to reach each constant value of sweat lactate elimination per unit before and after fatigue. The participants for whom the peak value exceed over the measurable range (a) and those for whom the value did not reach each constant value in either test (b–e) were excluded. A positive value indicates that the value of sweat lactate has reached the constant value sooner in Test 2 compared with Test 1.

^a^ *n* =17.

^b^ *n* =17.

^c^ *n* =14.

^d^ *n* =13.

^e^ *n* =11.

* Test 1 (before fatigue) versus Test 2 (after fatigue) using paired *t*‐test.

## 4 DISCUSSION

We conducted two consecutive constant workload exercise tests with rest intervals in 17 recreational athletes, providing information about the leftward shift of the sLA curve in the exercise after exhaustion. This novel finding indicated that the peak in the sweat lactate elimination curve during constant workload exercise was detected sooner with fatigue than without fatigue.

### 4.1 Fatigue from Test 1 to Test 2

The NRS is widely used for measuring fatigue in various persons, including those with spinal cord injury (Alschuler et al., 2013), hip and knee osteoarthritis (Ornetti et al., 2011), cancer (Schwartz et al., 2002), and rheumatoid arthritis (Nicklin et al., 2010). NRS is easy to administer and score, does not require extensive instrumentation, and is cost effective, indicating its applicability in the measurement of acute fatigue or perception of effort following fatigue caused by exercises, such as elbow flexion exercise (Lampropoulou & Nowicky, 2012) or side sliding exercise (Briem et al., 2017). In the present study, all participants performed pedaling exercise until exhaustion in Test 1 with the same relative load while there were wide range of TE, which mean that various metabolic pathways were guessed to contribute by participant. Nevertheless, all of them were fatigued during Test 2, which was shown by the significant increase in NRS at each time point in Test 2 compared with Test 1 (Figure 2) as a result. Previous reports have shown that fatigue, as indicated using NRS, also affects the muscular activity (Lampropoulou & Nowicky, 2012). Thus, the motor function of participants performing a pedaling exercise may have been fatigued after Test 1 as it required the participants to continue with the exercise until exhaustion in the present study.

### 4.2 Transition of sLA after fatigue

Recently, various types of sLA wearable sensors have been developed (Currano et al., 2018; Gao et al., 2016; Kai et al., 2018; Luo et al., 2018; Vinoth et al., 2021). These recent advances in wearable lactate sensors enable real‐time monitoring of sLA and capture detailed changes in the sLA value in response to changes in exercise intensity or over time. Taking advantage of this, Jia et al. (2013) reported that sLA increases with increased exercise intensity. In addition, our group clarified the applicability of detecting the lactate threshold based on real‐time lactate monitoring in sweat (Seki et al., 2021). Similar to previous reports, the present study used a sLA sensor to capture changes in sLA during constant‐load exercise and demonstrated that after fatigue, sLA rises sooner than before fatigue, that is, the lactate elimination curve shifts to the left after fatigue (Figures 3 and 4). This preliminarily result suggests that the sweat lactate elimination curve is different in the fatigue state. Further research may provide insight in the application of this curve to the evaluation for fatigue. As this method allows easy, real‐time evaluation of multiple players in a team, it may be beneficial to apply this method to condition management and injury prevention, for example, by comparing conditions of the different athletes during warm‐up on a game day compared to a rest day. It is difficult to estimate the cause of specific alterations in the sLA curve after fatigue because there is little evidence concerning changes in the sweat gland metabolism after fatigue. However, some physiological changes that induce a rise in sLA, such as changes in the autonomic nervous balance, hormones, acid–base equilibrium, and metabolic dynamics, can be evaluated (Alvear‐Ordenes et al., 2005; Benson et al., 1974; Quinton, 1987; Yamamoto et al., 1991). Further research is warranted to confirm this.

### 4.3 Limitation

This study had several limitations. First, we could not calculate an adequate sample size because there was no previous reference study that investigated the sLA curve transition. However, the statistical power of the paired *t*‐test for evaluating lactate curve shift was over 0.99 for all values, all of which were over 0.8, as recommended by Cohen (1992). Second, to cause exhaustion, we applied only a single workload to constant‐load exercise in this study. In addition to the comparison between the sLA curve in fatigued and non‐fatigued state conducted in this study, the similar experiment with various relative loads may provide further finding including possibility to detect quantitativity of fatigue using wearable sLA sensor. Further accumulation of data is need to this issue. Third, it is possible that the inability of our study to monitor certain intake parameters, such as intake of water, carbohydrates, and other nutrients, affected our findings, although intake of water was restricted within 3 hours before the experiment, according to a previous study (Pöchmüller et al., 2016). Increased muscle glycogens lead to increased glycometabolism (Richter & Galbo, 1986) and improved endurance performance (Lima‐Silva et al., 2009) during high‐intensity activities, similar to amino (Brooks, 1987; Matsumoto et al., 2009) or taurine (Takahashi et al., 2016). However, uncontrolled water intake may have little effect because we encouraged the participants to drink water before the test, and a previous report indicated that dehydration had no effect on the blood lactate acid curve (Van Schuylenbergh et al., 2005). Fourth, because this study included healthy college‐aged male individuals, further research is needed in females and young athletes based on a sweat functional difference between genders (Frye & Kamon, 1983; Wyndham et al., 1965) and age (Meyer et al., 2007). Fifth, it is possible that psychological factors may negatively affect the three participants who were unable to proceed to Test 2. Last, replacement of sensor chips during the rest period could have affected our results. Meanwhile, in vitro data shown in a previous study indicate that the chip‐to‐chip error is small up to values of 15 µA (Seki et al., 2021). Since all sweat lactate values for this study remained under 15 µA (Figures S3 and S4), it is considered that the effect caused by the replacement of chip sensors may be minor. Additionally, to relieve this effect further, we performed zero correction for the sweat lactate value using a baseline value.

## 5 CONCLUSION

In conclusion, we identified a leftward shift in the obtained sLA curve in constant‐load exercise after fatigue. This preliminary result suggests that the sweat lactate elimination curve is different in the fatigue state. Further research is needed to investigate this trend in various participants and its relationship with physiological factors in sweat glands.

## CONFLICT OF INTERESTS

Daisuke Nakashima is the president of Grace Imaging Inc. and hold shares in this company which sells lactic acid sensing equipment. Daisuke Nakashima was not involved in data acquisition and analysis.

## AUTHOR CONTRIBUTIONS

All authors contributed to the study conception and design. Material preparation and data collection were performed by Hiroki Okawara, Tomonori Sawada, Yuta Maeda, Shunsuke Minoji, and TM. Analysis was conducted by Hiroki Okawara, Tomonori Sawada, and Yoshinori Katsumata mainly and supported by Morio Matsumoto, Masaya Nakamura, and Takeo Nagura. The first draft of the manuscript was written by Hiroki Okawara and all authors commented on previous versions of the manuscript. All authors read and approved the final manuscript. Daisuke Nakashima is responsible for all work excluded data acquisition and analysis related to this submission as the corresponding author, who is supported by Yoshinori Katsumata for work of corresponding author partially.

## Supporting information



Fig S1

Fig S2

Fig S3

Fig S4

Fig S5

Fig S6

Table S1
