# Supplementary material for: Kinetic changes in sweat lactate following fatigue during constant workload exercise
Source: Physiol Rep. 2022 Jan 19;10(2):e15169. doi: 10.14814/phy2.15169 (PMC8767313; doi:10.14814/phy2.15169)

Supplementary Figure 1. The change of objective fatigue over time in test 1 and 2

This value of numerical rating scale with face rating scale significantly increased gradually over time in test 1 and 2; * $P < 0.05$, ** $p < 0.01$ using Friedman's test and Bonferroni method.

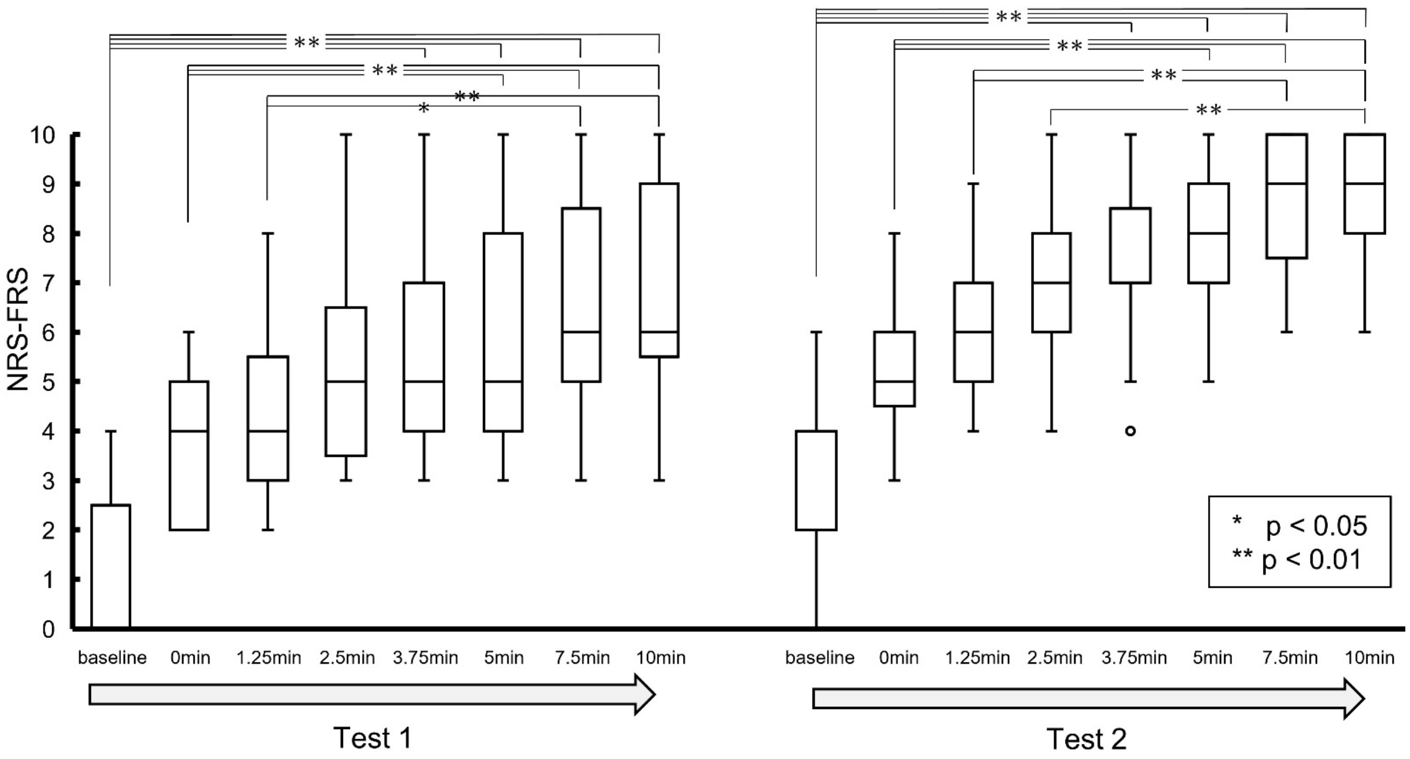

Supplement: Supplementary file 1 — Fig S1 [file PHY2-10-e15169-s005.pdf]
