# Supplementary material for: Kinetic changes in sweat lactate following fatigue during constant workload exercise
Source: Physiol Rep. 2022 Jan 19;10(2):e15169. doi: 10.14814/phy2.15169 (PMC8767313; doi:10.14814/phy2.15169)

Supplementary Figure 2. The trend of lactate in sweat obtained at arm during pedaling exercise at constant load before and after fatigue

This figure shows average data of lactate in sweat obtained at arm at each time point during pedaling exercise with constant workload in test 1 and 2. Sweat lactate exhibited sooner point to reach the peak value in test 2

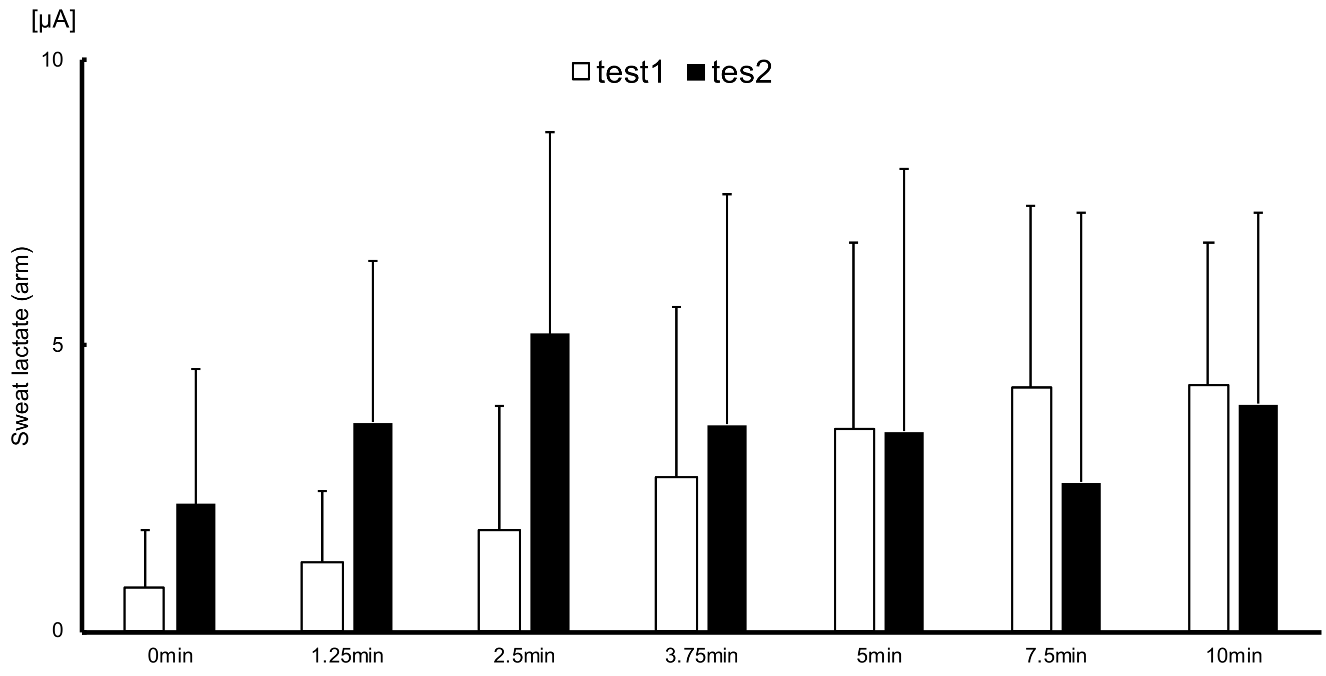

Supplement: Supplementary file 2 — Fig S2 [file PHY2-10-e15169-s001.pdf]
