# Supplementary material for: Kinetic changes in sweat lactate following fatigue during constant workload exercise
Source: Physiol Rep. 2022 Jan 19;10(2):e15169. doi: 10.14814/phy2.15169 (PMC8767313; doi:10.14814/phy2.15169)

Supplementary figure 4. Sweat rate obtained at head during constant load exercise

This figure shows sweat rate at each time point during pedaling exercise with constant workload in test 1 and 2.

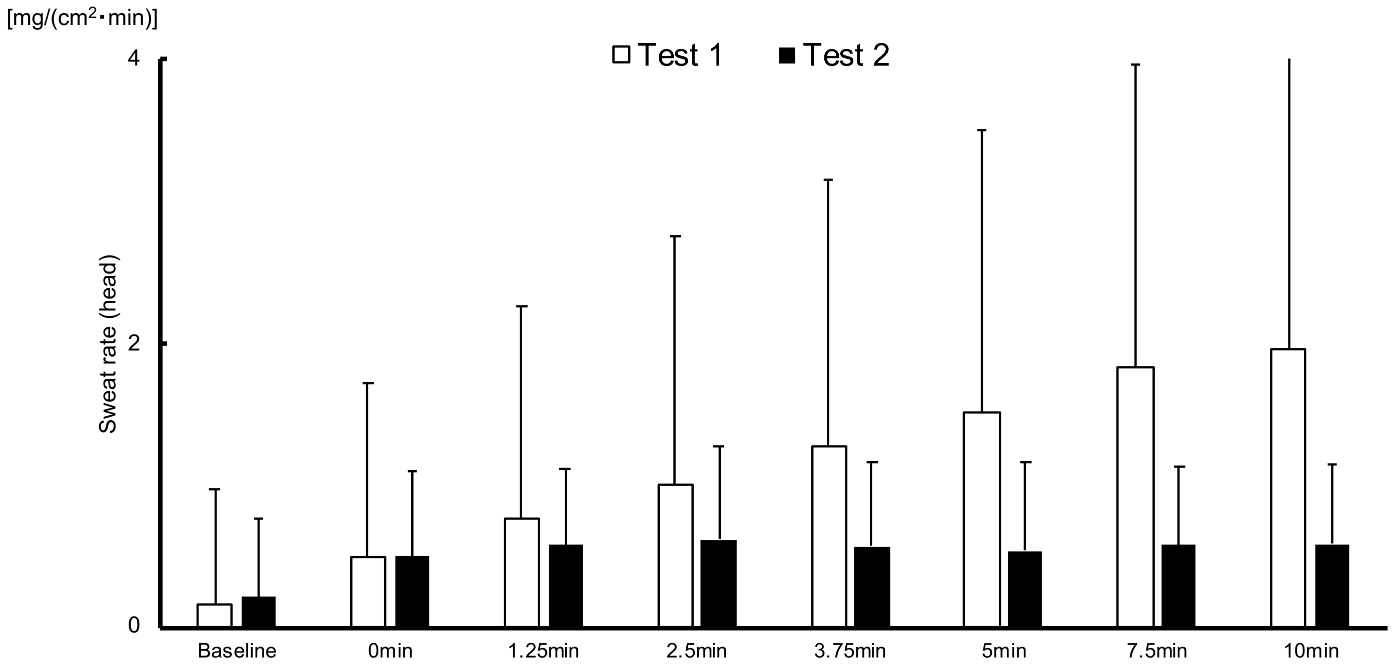

Supplement: Supplementary file 4 — Fig S4 [file PHY2-10-e15169-s003.pdf]
