# Supplementary material for: Kinetic changes in sweat lactate following fatigue during constant workload exercise
Source: Physiol Rep. 2022 Jan 19;10(2):e15169. doi: 10.14814/phy2.15169 (PMC8767313; doi:10.14814/phy2.15169)

**Supplementary Figure 6. The trend of sweat lactate elimination per unit area (at head) during pedaling exercise at constant load before and after fatigue**

This figure shows each average data of sweat lactate elimination per unit area at head (filled circle) at each time point during pedaling exercise with constant workload in test 1 and 2. Sweat lactate exhibited sooner point to reach the peak value in test 2 contrary to similar trends of linear increasing it in test 1.

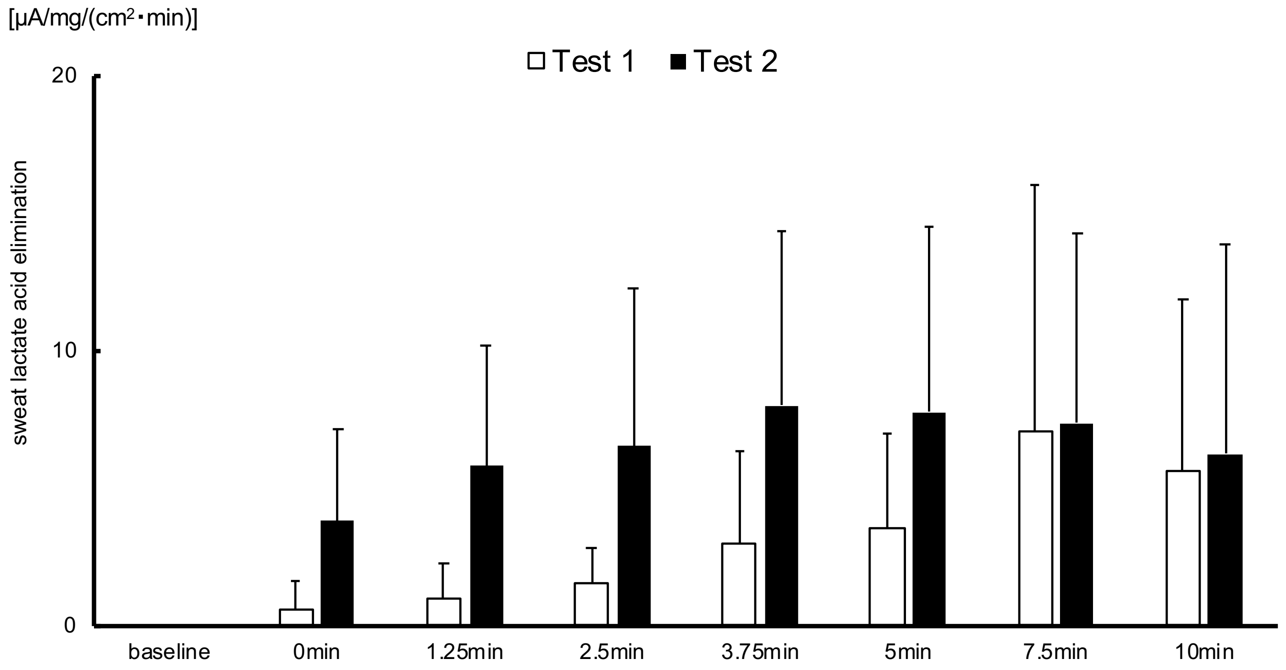

Supplement: Supplementary file 6 — Fig S6 [file PHY2-10-e15169-s007.pdf]
