# Supplementary material for: Kinetic changes in sweat lactate following fatigue during constant workload exercise
Source: Physiol Rep. 2022 Jan 19;10(2):e15169. doi: 10.14814/phy2.15169 (PMC8767313; doi:10.14814/phy2.15169)
Supplement: Supplementary file 7 — Table S1 [file PHY2-10-e15169-s002.pdf]

Supplementary Table 1. Individual data of characteristics and peak power

This table shows individual data of peak power and 25 % power of peak power obtained from Wingate test using resistance was applied of 11% of body weight. Abbreviation; Peak Power$_{25\%}$ = 25 % power of peak power

No	Age [yr]	Height [cm]	Weight [kg]	BMI	Body Fat % [%]	Fat mass [kg]	Lean Bodt Mass [kg]	Muscle mass [kg]	Total Body Water [kg]	Body Water %	sports	Peak power [W]	Peak Power$_{25\%}$ [W]
1	21	168.8	57.3	20.1	12.0	6.90	50.4	47.8	35.4	61.8	Swimming	409	102
2	20	170.2	59.1	20.4	14.0	8.30	50.8	48.2	34.6	58.5	Tennis	672	168
3	21	174.9	60.9	19.9	16.6	10.10	50.8	48.2	33.9	55.7	Golf	658	165
4	20	163.1	47.1	17.7	7.5	3.50	43.6	41.3	30.8	65.4	Swimming	511	128
5	19	169.6	64.7	22.5	18.1	11.70	53.0	50.2	35.1	54.3	Basketball	679	170
6	20	164.5	57.4	21.2	21.0	12.10	45.3	42.9	29.2	50.9	Golf	371	93
7	21	165.1	48.8	17.9	10.8	5.30	43.5	41.2	30.0	61.5	Ice hockey	493	123
8	21	170.3	64.1	22.1	16.3	10.40	53.7	50.9	37.8	50.0	Basketball	610	153
9	21	181.9	71.8	21.7	19.4	13.90	57.9	54.9	39.2	54.6	Tennis	810	203
10	20	173.8	59.8	19.8	13.6	8.10	51.7	49.0	35.0	58.5	Badminton	691	173
11	21	181.7	75.9	23.0	20.1	15.30	60.6	57.5	41.2	54.3	Boat	644	161
12	22	164.8	61.4	22.6	17.9	11.00	50.4	47.6	36.5	59.4	Volleyball	528	132
13	21	173.6	71.4	23.7	24.8	17.70	53.7	50.9	35.6	49.9	Swimming	639	160
14	20	175.8	70.8	22.9	16.8	11.90	58.9	55.8	41.7	58.9	Diging	635	159
15	19	170.2	67.2	23.2	15.9	10.70	56.5	53.6	40.5	60.3	Handball	744	186
16	22	176.8	68.8	22.00	16.4	11.30	57.5	54.5	40.5	58.9	Soccer	675	169
17	21	168.2	64.2	22.7	14.0	9.00	55.2	52.3	40.7	63.4	Soccer	830	208
